# Supplementary material for: Rare Filaggrin Variants Are Associated with Pustular Skin Diseases in Asians
Source: Int J Mol Sci. 2024 Jun 12;25(12):6466. doi: 10.3390/ijms25126466 (PMC11203790; doi:10.3390/ijms25126466)
Supplement: Supplementary file 1 [file ijms-25-06466-s001.zip › ijms-3034442-supplementary.pdf]

SUPPLEMENTARY DATA

Rare filaggrin variants segregate with pustular skin diseases in Asians.

Luca Lo Piccolo[1], Wasinee Wongkummool[1], Phatcharida Jantaree[1], Teerada Daroontum[2], Suteeraporn Chaowattanapanit[3], Charoen Choonhakarn[3], Warayuwadee Amornpinyo[4], Romanee Chaiwarith[5], Salin Kiratikanon[6], Rujira Rujiwetpongstorn[6], Napatra Tovanabutra[6], Siri Chiewchanvit[6], Chumpol Ngamphiw[7], Worrachet Intachai[8], Piranit Kantaputra[8,9], Mati Chuamanochan[6]

[1] Centre of Multidisciplinary Technology for Advanced Medicine (CMUTEAM), Faculty of Medicine, Chiang Mai University, Chiang Mai, 50200, Thailand.
[2] Department of Pathology, Faculty of Medicine, Chiang Mai University, Chiang Mai, 50200, Thailand
[3]Division of Dermatology, Department of Medicine, Faculty of Medicine, Khon Kaen University, Khon Kaen, 40002, Thailand
[4] Division of Dermatology, Department of Internal Medicine, Khon Kaen Hospital, Khon Kaen, 40002, Thailand
[5] Division of Infectious Diseases and Tropical Medicine, Department of Internal Medicine, Chiang Mai University, Chiang Mai, 50200, Thailand.
[6] Division of Dermatology, Department of Internal Medicine, Faculty of Medicine, Chiang Mai University, Chiang Mai 50200, Thailand.
[7] National Center for Genetic Engineering and Biotechnology, National Science and Technology Developmemt Agency, Pathum Thani, 12120, Thailand.[8] Center of Excellence in Medical Genetics Research, Faculty of Dentistry, Chiang Mai University, Chiang Mai 50200, Thailand
[9] Division of Pediatric Dentistry, Department of Orthodontics and Pediatric Dentistry, Faculty of Dentistry, Chiang Mai University, Chiang Mai 50200, Thailand.

*** Correspondence:**
Mati Chuamanochan
Division of Dermatology,
Department of Internal Medicine,
Faculty of Medicine, Chiang Mai University,
Chiang Mai 50200, Thailand
Email: mati.c@cmu.ac.th

Supplementary Figure S1 Whole Exome Sequencing and bioinformatics analysis. The diagram shows the bioinformatics analysis to identify the pathogenic variants and genes associated with pustule skin diseases from 17 AOID and 24 GPP whole exome sequencings data.

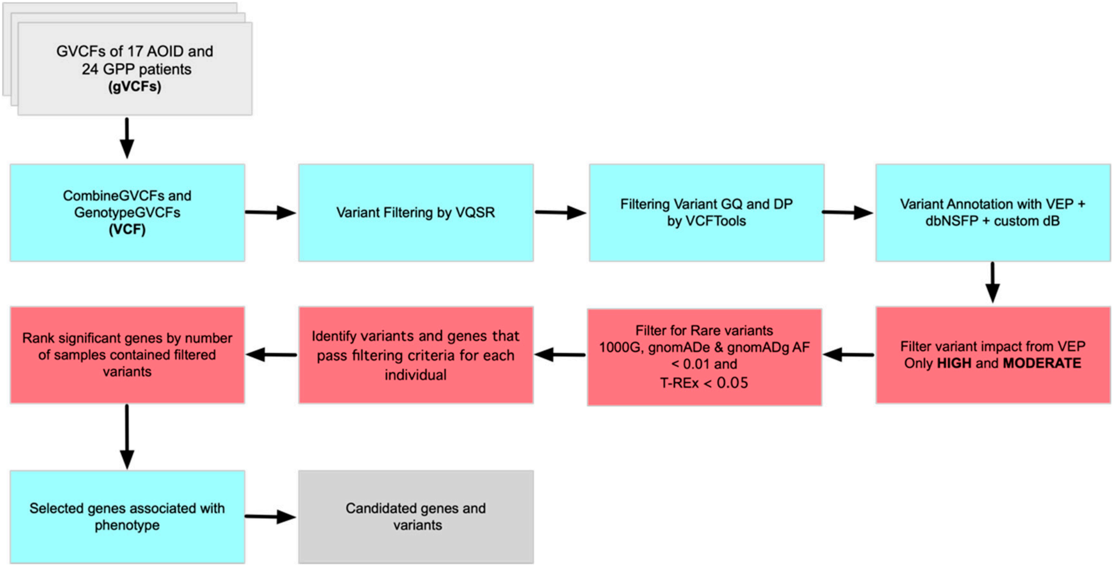

Supplementary Figure S2

High resolution imaging of formalin-fixed, paraffin-embedded normal healthy skin stained with Filaggrin antibody (a representative picture is shown out of 10 healthy individual's skin biopsy). Filaggrin is expressed only in well-differentiated keratinized epithelial cells including hair follicles. A) Normal healthy control skin (4X). B) Normal healthy control skin (8X). AOID-PR patients and PP patients displayed reduced Filaggrin immunoreactivity compared to that of normal healthy controls. C) AOID-PR-FLG-2797 (8X). D) AOID-PR-FLG-2925 (8X). E) AOID-PR-FLG-2935 (8X). F, AOID-PR-FLG-2926 (8X). G, AOID-FLG-2923 (8X). H, AOID-PR-FLG-WT (8X) (three technical replicates have been performed for each patient's biopsy).

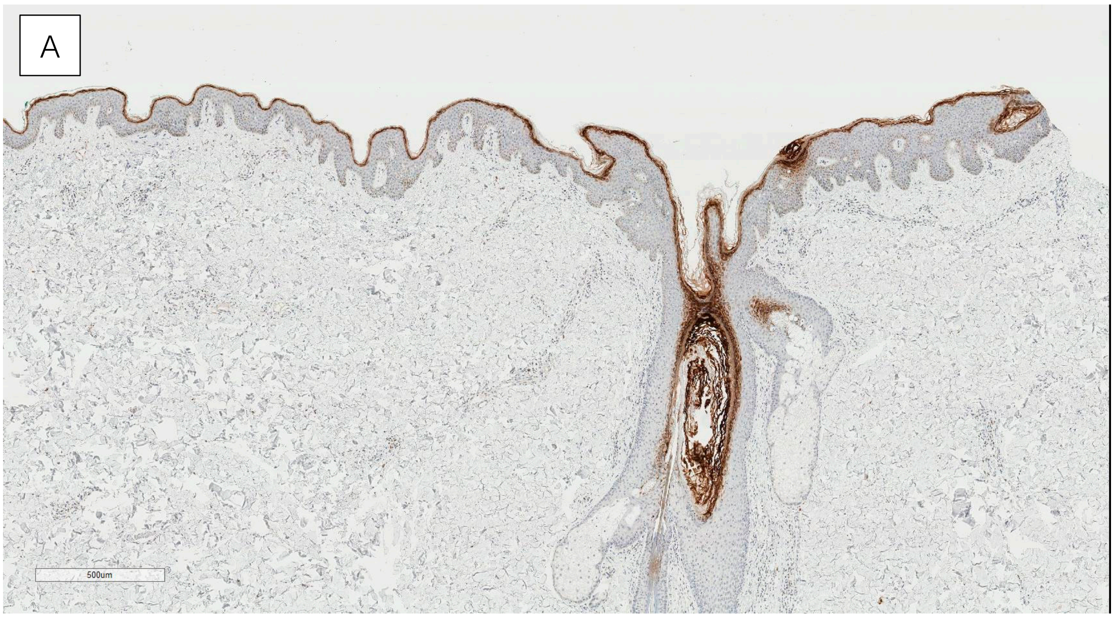

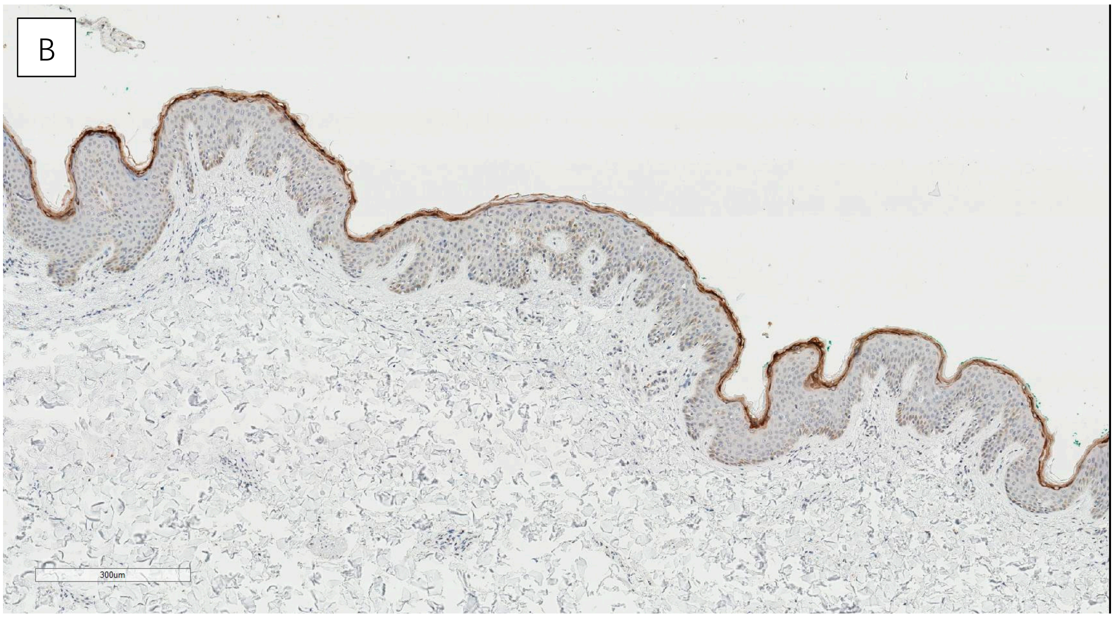
B
300um

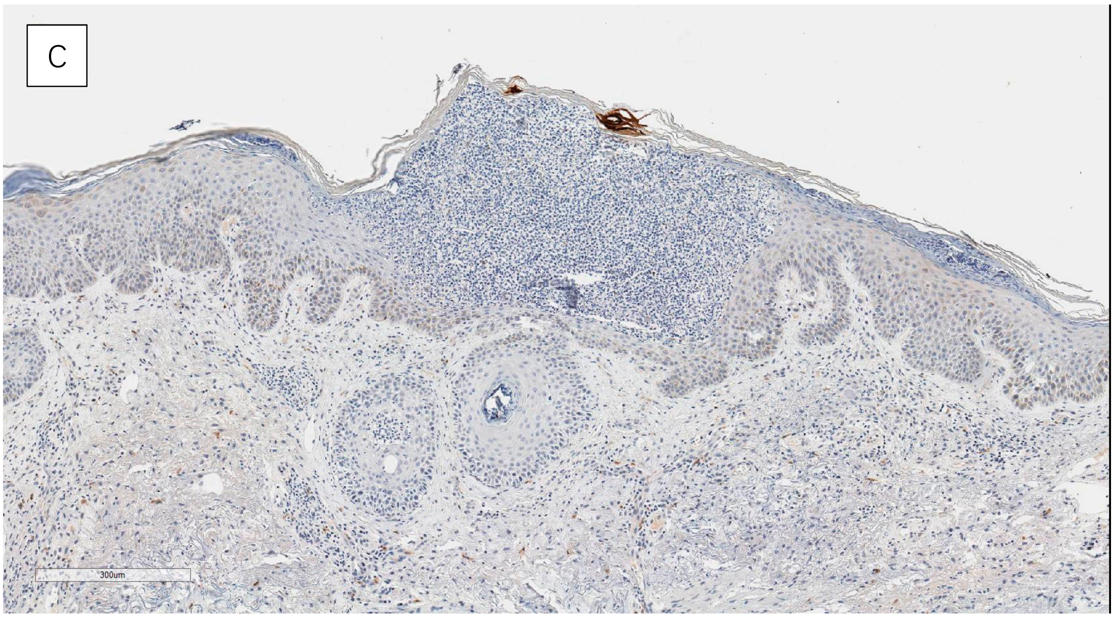
C
300um

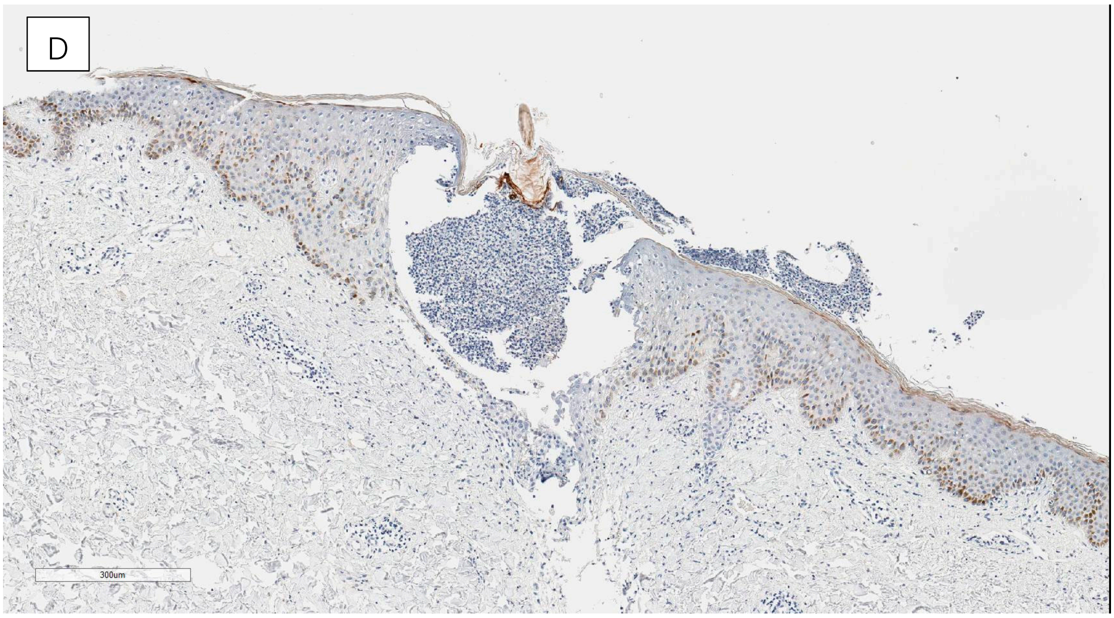
D
300um

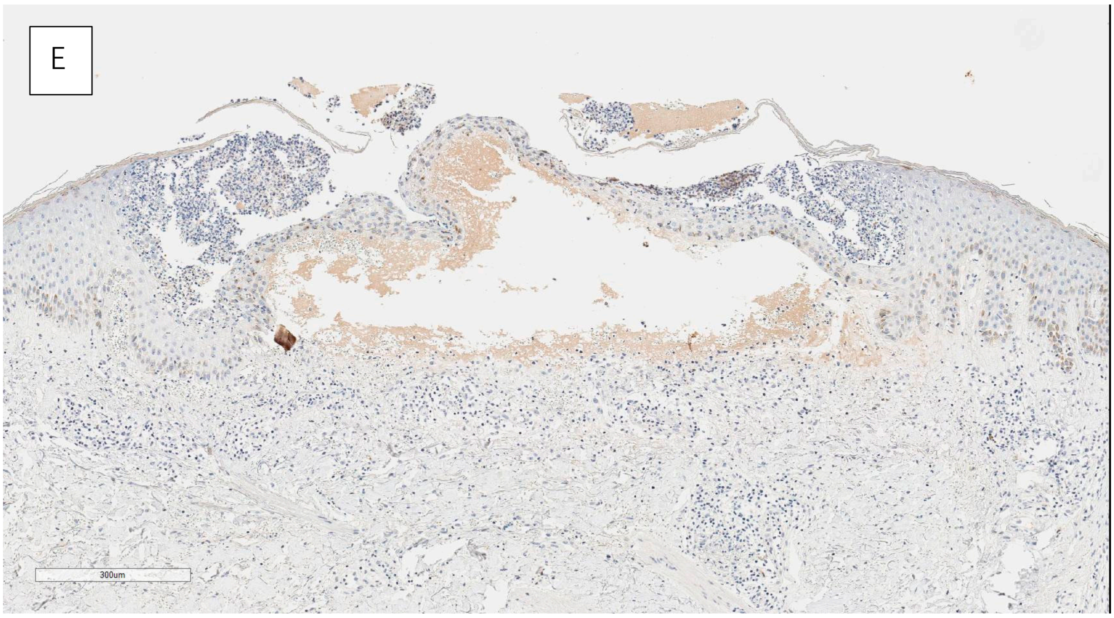
E
300um

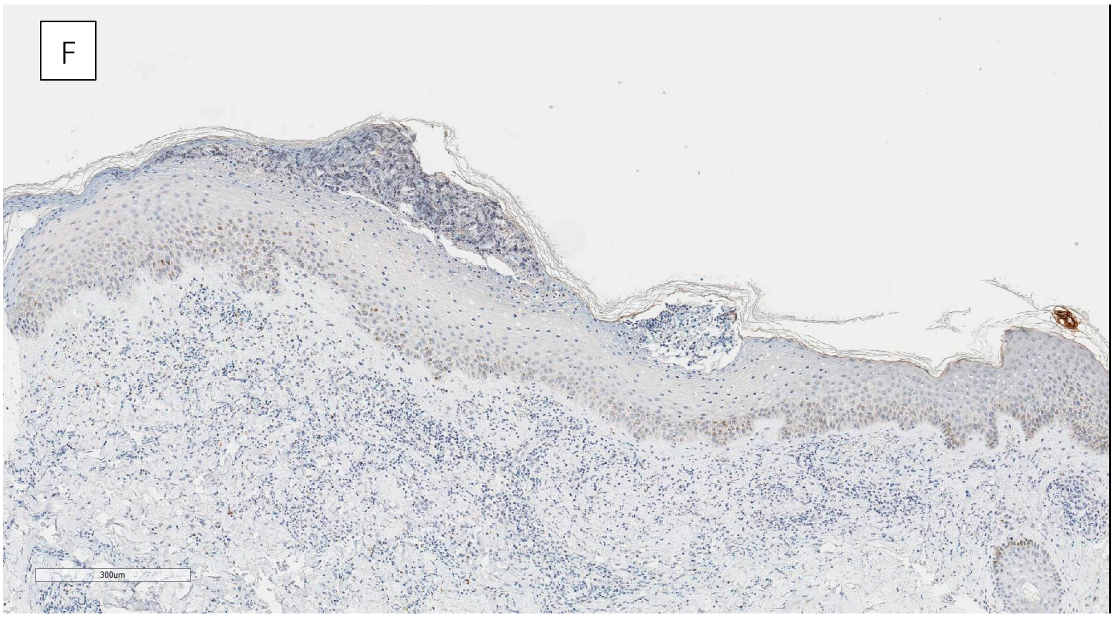
F
300um

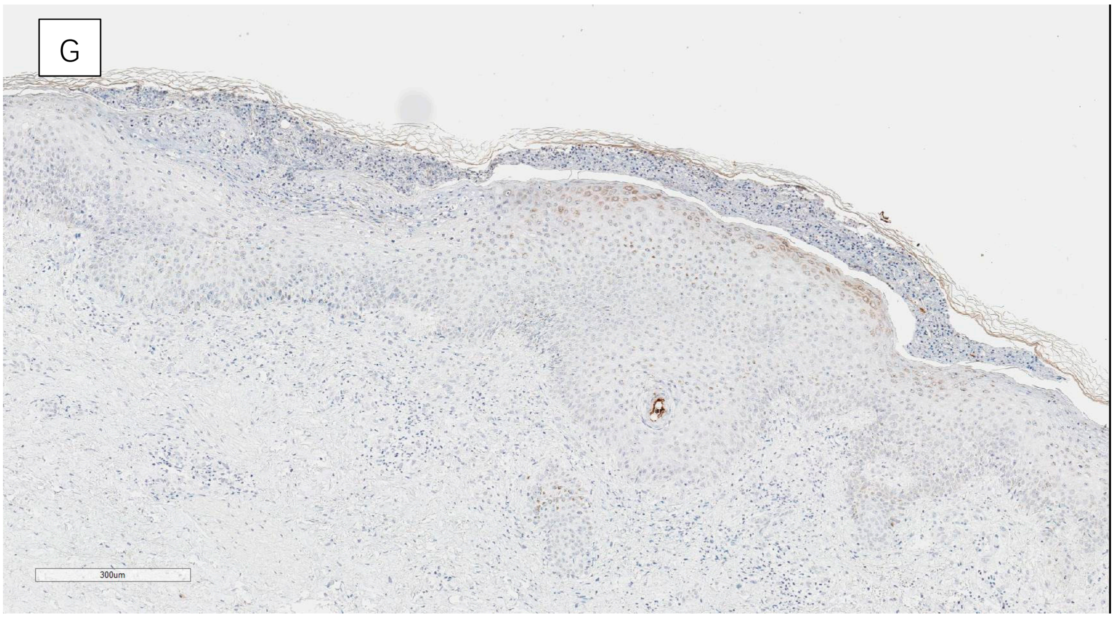
G
300um

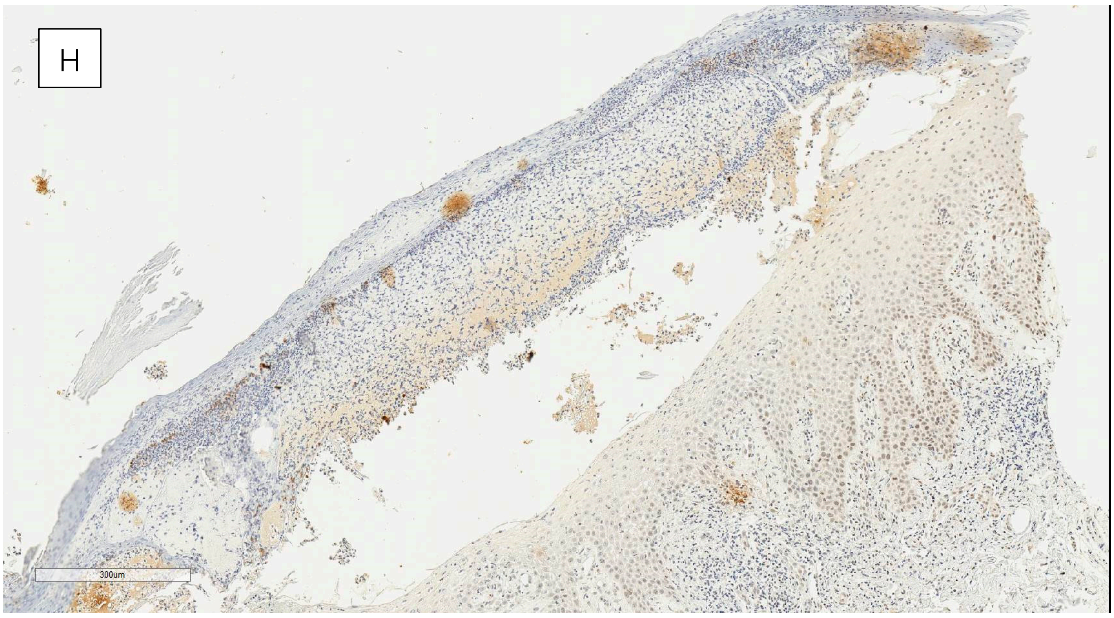
H
300um

Supplementary Table S1 Details of Filaggrin FLG variants identified in AOID-PR patients.

Gene	SNP	Pos	Sample	Consequence	Codons	Impact	Clinical classification
FLG	n.r.	152275655	2935	stop_gained	Gga/Tga	HIGH	n.a.
FLG	rs145079750	152275876	2925	missense	cGt/cAt	MODERATE	LB
FLG	rs533740963	152278606	2951	missense	gCt/gTt	MODERATE	B
FLG	rs542799026	152279245	2804	stop_gained	tCa/tGa	HIGH	P
FLG	rs1285949984	152281599	2951	missense	gaC/gaA	MODERATE	LB
FLG	rs200551704	152282941	2797	missense	cGa/cAa	MODERATE	LB
FLG	rs112252908	152283173	2787	missense	Aac/Gac	MODERATE	LB
FLG	rs527894804	152283178	2787	missense	gTc/gCc	MODERATE	LB
FLG	rs139061200	152283193	2787	missense	tCc/tAc	MODERATE	B
FLG	rs547196696	152284540	2911	missense	cGc/cAc	MODERATE	LB
FLG	rs201661720	152284783	2926	missense	tCg/tGg	MODERATE	B

n.r = not reported; n.a.= not available; B = benign; LB = likely benign; P = pathogenic

Supplementary Table S2 Details of Filaggrin FLG variants identified in PP patients

Gene	SNP	Pos	Sample	Consequence	Codons	Impact	Clinical classification
FLG	COSV64244543	152275780	2923	missense	tCc/tAc	MODERATE	LB
FLG	rs769188915	152276113	2886	missense	gCg/gTg	MODERATE	n.a.
FLG	rs200423945	152279527	2922	missense	gAc/gGc	MODERATE	n.a.
FLG	rs555272052	152280043	2900	missense	gGa/gCa	MODERATE	n.a.
FLG	rs780793108	152280956	2907	missense	Gac/Tac	MODERATE	n.a.
FLG	rs142983961	152280963	2923	missense	gaG/gaC	MODERATE	n.a.
FLG	rs145675213	152282956	2882	missense	cGc/cAc	MODERATE	n.a.
FLG	rs532746197	152283799	2910	missense	tCt/tAt	MODERATE	n.a.
FLG	rs143643121	152284441	2922	missense	gCt/gTt	MODERATE	LB
FLG	rs369325094	152285326	2916	missense	aAa/aTa	MODERATE	n.a.

n.a.= not available; LB = likely benign

Supplementary Table S3 Details of Filaggrin FLG3 variants identified in AOID-PR patients.

Gene	SNP	Pos	Carrier	Consequence	Codons	Impact	Clinical classification
FLG3	n.r.	152185616	2904	frameshift	ctCAgt/ctgt	HIGH	n.a.
FLG3	rs753299485	152187194	2925	missense	cGa/cAa	MODERATE	n.a.
FLG3	rs768542886	152188769	2803	missense	aGc/aAc	MODERATE	n.a.
FLG3	rs758134162	152191178	2797	missense	aGc/aCc	MODERATE	n.a.
FLG3	rs200674313	152191206	2898	missense	Tca/Cca	MODERATE	n.a.
FLG3	rs141263661	152191578	2914, 2938	stop_gained	Cga/Tga	HIGH	n.a.
FLG3	rs765957331	152192103	2914	missense	Gat/Tat	MODERATE	n.a.

n.r = not reported; n.a.= not available

Supplementary Table S4 Details of Filaggrin FLG3 variants identified in PP patients

Gene	SNP	Pos	Sample	Consequence	Codons	Impact	Clinical classification
HRNR	rs753301905	152187069	2922	missense	Tct/Cct	MODERATE	n.a.
HRNR	rs1334755098	152187437	2895	missense	gTc/gCc	MODERATE	n.a.
HRNR	rs750289349	152188548	2877	missense	Ggt/Agt	MODERATE	n.a.
HRNR	rs755669550	152188605	2933	missense	Cga/Gga	MODERATE	n.a.
HRNR	rs748246449	152188719	2895	missense	Gag/Cag	MODERATE	n.a.
HRNR	rs200844006	152190330	2922	missense	Ggc/Agc	MODERATE	n.a.
HRNR	rs769962080	152192465	2921	missense	cGa/cAa	MODERATE	n.a.
HRNR	rs566122590	152192966	2905	missense	aCg/aGg	MODERATE	n.a.
HRNR	rs368737363	152193248	2937	missense	cAt/cGt	MODERATE	n.a.

n.a.= not available
